# Outbreak of chickenpox in a refugee camp of northern Thailand

**DOI:** 10.1186/1752-1505-4-4

**Published:** 2010-02-22

**Authors:** Yusuke Shimakawa, Olivier Camélique, Koya Ariyoshi

**Affiliations:** 1 gCOE program, Institute of Tropical Medicine (Nekken), Nagasaki University, 1-12-4, Sakamoto, Nagasaki-shi, 852-8523, Japan; 2 Médecins Sans Frontières, 8 rue Saint Sabin, 75011, Paris, France

## Abstract

Although chickenpox is a generally mild, self-limited illness of children, it can cause fatal disease in adults. Accumulating reports from tropical countries showed a high prevalence of seronegativity among the adults, implying that varicella diseases could become a heavy burden in tropical countries. However, in the situation of humanitarian emergencies in tropical areas, chickenpox has largely been ignored as a serious communicable disease, due to lack of data regarding varicella mortality and hospital admissions in such a context. This is the first report describing an outbreak of chickenpox in a refugee camp of tropical region. In 2008, we experienced a varicella outbreak in ethnic Lao Hmong refugee camp in Phetchabun Province, northern Thailand. The attack rate was 4.0% (309/7,815) and this caused 3 hospitalizations including one who developed severe varicella pneumonia with respiratory failure. All hospitalizations were exclusively seen in adults, and the proportion of patients ≥15 years old was 13.6% (42/309). Because less exposure to varicella-zoster virus due to low population density has previously been suggested to be one of the reasons behind higher prevalence of susceptible adults in tropics, the influx of displaced people from rural areas to a densely populated asylum might result in many severe adult cases once a varicella outbreak occurs. Control interventions such as vaccination should be considered even in refugee camp, if the confluence of the risk factors present in this situation.

## Findings

Although varicella occurs universally, its epidemiology is remarkably different in tropical and temperate areas [1]. In temperate countries, more than 90% of people are infected before adolescence [2], whereas in tropical regions varicella tends to occur at a later age causing many adult cases, suggested by the reports, demonstrating a high prevalence of varicella seronegativity among adolescents and adults [1,3-6].

Adult varicella patients are known to develop severe disease with a higher rate of complications than children [7], and pregnant women with primary varicella-zoster virus (VZV) infection are at risk of transmitting the infection to their unborn child causing congenital varicella syndrome. Although these factors indicate that the health burden attributable to varicella disease in tropical settings is much heavier than previously assumed [1], data on mortality and hospital admissions from the tropics are sparse [2]. Here, we report the first description of a varicella outbreak in a refugee camp in a tropical region. 

The international medical humanitarian organization Médecins Sans Frontières (MSF) began providing medical and logistic aid to ethnic Lao Hmong refugees in Phetchabun Province, northern Thailand in July 2005, and opened the sole outpatient clinic in this camp. People were confined to a guarded, barbed-wire enclosed camp controlled by the Thai military, and one household unit consisting of an average of 5 to 6 persons living in a small barracks. Barracks are clustered close together in a small area of 20 hectares. The total registered population in February 2008 was 7,815, including 1,930 children <5 years old. Although the majority recently fled from Laos, an estimated 1,000 Hmong were from a former refugee camp in central Thailand [8]. On the basis of MSF data in 2007, the average monthly crude mortality rate at the camp was 1.89 per 10,000 persons. Although there is no information on the overall prevalence of HIV infection, no cases were detected since the introduction of routine voluntary testing for pregnant women. As there was no inpatient facility inside the camp, seriously ill individuals were referred to the district hospital.

**Figure 1:**
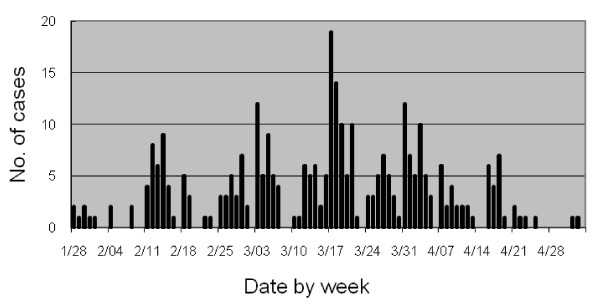
**Epidemic curve for varicella outbreak**. Number of varicella cases by date of diagnosis in the Lao Hmong refugee camp, from Jan 28 to May 3, 2008.

During the fifth week of January 2008, the incidence of varicella started to increase in the camp. Between January 28 and May 3, there were 309 cases of varicella (Figure 1), which was defined as an acute onset of diffuse maculo-papulovesicular rash without other apparent causes [9]. Overall community attack rate was 4.0% (309/7,815): 8.5% (164/1,930) and 2.5% (145/5,885) for those aged <5 years and ≥5 years, respectively. Median age was 4 years, ranging from 3 months to 53 years, and 42 (13.6%) patients were ≥15 years old. Among 165 (53.4%) female patients, five were pregnant. No severe complications occurred among the patients <15 years old, except 2 children with cellulitis who were treated on an outpatient basis. However, among the 42 patients ≥15 years old, 3 (7.1%) were admitted to the district hospital. They were all previously healthy: a 30-year-old man with severe dehydration, a 21-year-old pregnant woman with varicella pneumonia, and a 53-year-old man with varicella pneumonia followed by respiratory failure which required mechanical ventilation and admission to ICU. They were discharged without sequelae. All pregnant women who contracted varicella were followed-up until delivery, with no documented complications including birth defects. There were no particular clusters of barracks at higher risk of infection, and the isolation of patients as control measure was not feasible in this context. Only symptomatic treatment and antibiotics were available for case management.

This outbreak highlights two important findings. While the majority of patients were children, we found the proportion of patients ≥15 years old to total number of patients was higher than in temperate countries: 3% in the United States during the prevaccine era [7], 8% in France [10] and <1% in Japan [11]. Furthermore, all the hospitalizations were exclusively seen in those aged ≥15 years and the rate of hospitalizations per 1,000 cases was 9.7 (95% CI, 2.0-28.4). This was also higher than those reported from temperate countries: 1.2 in the United States [7] and 4.7 in France [10].

The exact mechanism of such an epidemiological variation in the tropics is largely unknown. Nevertheless, proposed explanations included interference with VZV transmission from high temperature [3,5], less opportunity to contract VZV during childhood in rural areas [4,5], competition with other viral pathogens [12], and cross-antigenicity between VZV and herpes simplex virus [13]. As most of Hmong in this camp recently fled from the rural areas of Laos, one might speculate that many reached adulthood without coming into contact with VZV, and contracted varicella subsequently to their settlement in the camp of high population density. Although there were no deaths, two adult patients with varicella pneumonia were a substantial health burden for this camp. These indicate that in tropical regions, the influx of displaced people from rural areas to a densely populated asylum might result in many severe adult cases once a varicella outbreak occurs. Control interventions such as vaccination should be considered even in refugee camp, if the confluence of the risk factors present in this situation.

## Competing interests

The authors declare that they have no competing interests.

## Authors' contributions

YS was a field doctor at this refugee camp, and collected all of the data as part of his routine work. OC was a medical coordinator of this camp, and supervised work of YS. KA participated in the interpretation of the results. OC and KA participated in the critical revision of the manuscript. All authors read and approved the final manuscript.
